# Optimizing Automated Hematoma Expansion Classification from Baseline and Follow-Up Head Computed Tomography

**DOI:** 10.3390/app15010111

**Published:** 2024-12-27

**Authors:** Anh T. Tran, Dmitriy Desser, Tal Zeevi, Gaby Abou Karam, Julia Zietz, Andrea Dell’Orco, Min-Chiun Chen, Ajay Malhotra, Adnan I. Qureshi, Santosh B. Murthy, Shahram Majidi, Guido J. Falcone, Kevin N. Sheth, Jawed Nawabi, Seyedmehdi Payabvash

**Affiliations:** 1 Department of Radiology and Biomedical Imaging, Yale School of Medicine, New Haven, CT 06519, USA; 2 Department of Neuroradiology, Charité—Universitätsmedizin Berlin, Humboldt-Universität Zu Berlin, Freie Universität Berlin, Berlin Institute of Health, 10117 Berlin, Germany; 3 Zeenat Qureshi Stroke Institute and Department of Neurology, University of Missouri, Columbia, MO 65201, USA; 4 Department of Neurology, Weill Cornell School of Medicine, New York, NY 10065, USA; 5 Department of Neurosurgery, Mount Sinai School of Medicine, New York, NY 10029, USA; 6 Department of Neurology, Yale School of Medicine, New Haven, CT 06510, USA; 7 Department of Radiology, Columbia University Medical Center, New York, NY 10032, USA

**Keywords:** intracerebral hemorrhage, hematoma expansion, convolution neural network, segmentation, classification, ground truth generation, high sensitivity model

## Abstract

Hematoma expansion (HE) is an independent predictor of poor outcomes and a modifiable treatment target in intracerebral hemorrhage (ICH). Evaluating HE in large datasets requires segmentation of hematomas on admission and follow-up CT scans, a process that is time-consuming and labor-intensive in large-scale studies. Automated segmentation of hematomas can expedite this process; however, cumulative errors from segmentation on admission and follow-up scans can hamper accurate HE classification. In this study, we combined a tandem deep-learning classification model with automated segmentation to generate probability measures for false HE classifications. With this strategy, we can limit expert review of automated hematoma segmentations to a subset of the dataset, tailored to the research team’s preferred sensitivity or specificity thresholds and their tolerance for false-positive versus false-negative results. We utilized three separate multicentric cohorts for cross-validation/training, internal testing, and external validation (*n* = 2261) to develop and test a pipeline for automated hematoma segmentation and to generate ground truth binary HE annotations (≥3, ≥6, ≥9, and ≥12.5 mL). Applying a 95% sensitivity threshold for HE classification showed a practical and efficient strategy for HE annotation in large ICH datasets. This threshold excluded 47–88% of test-negative predictions from expert review of automated segmentations for different HE definitions, with less than 2% false-negative misclassification in both internal and external validation cohorts. Our pipeline offers a time-efficient and optimizable method for generating ground truth HE classifications in large ICH datasets, reducing the burden of expert review of automated hematoma segmentations while minimizing misclassification rate.

## 1. Introduction

Spontaneous intracerebral hemorrhage (ICH), or hemorrhagic stroke, is the most disabling form of stroke with a 40% mortality rate and 80% of survivors failing to regain functional independence [1]. The expansion of the initial hematoma volume affects more than one-third of patients and is associated with worse outcomes and increased mortality [2–7]. Identification of ICH patients at risk of hematoma expansion (HE) as a modifiable risk factor can guide targeted interventions, such as intensive blood pressure reduction, administration of hemostatic agents, or surgical evacuation of small hematomas at risk of expansion [8].

HE is commonly defined by binarizing the ICH volume increase from baseline to follow-up scans [9–12]. Increasing the expansion threshold—for example, from ≥3 mL to ≥6 mL or ≥12.5 mL HE—is associated with higher specificity and positive predictive value for poor outcomes and mortality [13]. Quantifying hematoma volumes on baseline and follow-up brain scans is the first step in identifying which ICH patients had HE. Manual segmentation of hematomas on brain scans by expert reviewers remains the gold standard for accurate volume quantification and determination of the ground truth in HE classification. However, this process is time-consuming, labor-intensive, and prone to inter-observer variability, making it impractical for large-scale data analysis. To address these challenges, automated segmentation tools are required to streamline the ICH segmentation process and HE classification, facilitating faster, more consistent, and scalable analysis of large ICH datasets.

Head CT scans are widely available, fast, and highly sensitive for acute brain hemorrhage and thus are the first line of imaging in an emergency setting when ICH is suspected. Many research groups have developed automated models for segmentation of ICH on head CT scans [14–20]. These models usually use U-shaped convolutional neural networks (CNNs) to automatically identify and delineate hemorrhagic lesions on head CT. Although automated ICH segmentation tools in head CTs are highly accurate [16–20], they are still subject to a degree of error. When quantifying hematoma volumes in two separate scans, such as baseline and follow-up head CTs, even small segmentation errors can accumulate. This compounding of errors affects the accurate quantification of hematoma volume changes from baseline to follow-up scan, leading to potential HE misclassification. A practical approach for accurate classification of HE in large datasets is to focus expert visual review only on subjects where cumulative errors in the segmentation of baseline and follow-up hematoma could be large enough to cause misclassification.

Mitigating cumulative errors from hematoma segmentation on baseline and follow-up scans for HE classification can be challenging, since different factors may introduce biases in segmentations of hematomas in these two separate scans. Correcting these errors based solely on extracted volume measurements may be inadequate, as volume data alone lack direct contextual information about the original scans. To address this issue, we propose a deep learning model that integrates segmentation masks with the original scans to directly classify each subject’s HE status.

**Figure 1.**
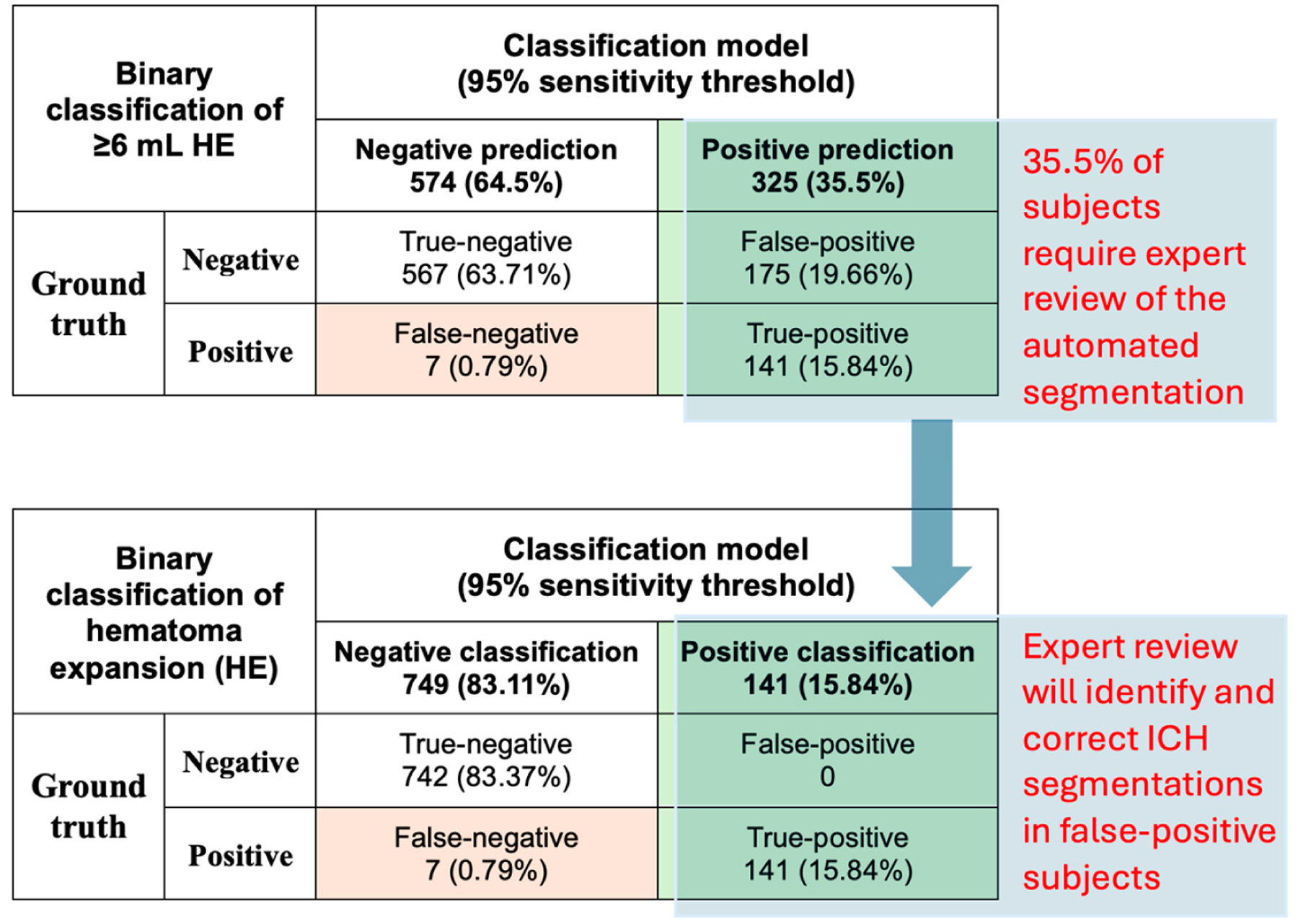
An example of an HE classification workflow with a high-sensitivity (95%) threshold classification. Combined segmentation and classification pipeline identifies the majority of subjects with HE (141 out of 148, 95.2%), and expert review of automated segmentations is limited to 35.5% of the subjects, correcting false-positive cases. This process results in 99.21% accurate HE classification in the whole dataset, with a final 0.7% false-negative rate. Notably, expert reviewers spend only a third of the time required for examining segmentations in the entire dataset, by focusing on test positive subjects, significantly improving efficiency. The approach is practical and efficient for generating ground truth annotations of HE in large ICH datasets.

Recognizing the difficulty of achieving absolute accuracy, we suggest using a high-sensitivity threshold for HE classification to minimize false-negative rates while limiting the number of test-positive cases requiring expert review of segmentation. In such a scenario, a highly sensitive model can accurately identify nearly all subjects with HE while reliably excluding those without HE, limiting the need for expert review to test-positive cases to correct potential false-positive instances. As shown in the example in Figure 1, by limiting expert review to approximately one-third of the dataset (35.5%), we achieved over 99% accuracy in HE classification, with a final false-negative rate of only 0.79%. Notably, this pipeline can be optimized for either high-sensitivity or high-specificity classifications, depending on the research team’s preference, to minimize false-negative or false-positive misclassifications, respectively. While not perfect, this strategy offers a practical and scalable solution for generating ground truth HE annotations in large ICH datasets. It minimizes the need for expert review to a small subset of scans while ensuring minimal misclassifications, providing a time-efficient and effective approach for large-scale studies.

In this study, we utilized three separate multicentric cohorts for training/cross-validation, as well as internal and external validation of our models and pipelines. We present the results from various classification strategies and sensitivity/specificity thresholds. All final models are publicly shared on GitHub.

## 2. Materials and Methods

### 2.1. Patients’ Cohorts

Three different datasets were used in this study: (1) the Yale dataset: A cohort of consecutive ICH patients who were admitted to Yale Comprehensive Stroke Center, New Haven, CT, USA, from August 2014 to November 2023 [3]; (2) the Antihypertensive Treatment of Cerebral Hemorrhage (ATACH-2) trial dataset: A multicentric cohort of patients enrolled in the ATACH-2 randomized trial who had primary acute ICH within 4.5 h of onset, a baseline hematoma volume < 60 mL, and at least one systolic blood pressure > 180 mm Hg, conducted across 110 sites in six countries: the United States, Japan, China, Taiwan, South Korea, and Germany [21]; and (3) the Charité dataset: A stroke center cohort of consecutive ICH patients admitted to the Charité University Hospital in Berlin, Germany [16]. The study included patients aged 18 years or older diagnosed with primary spontaneous ICH and available baseline and follow-up non-contrast head CT scans. Patients were excluded if they had significant imaging artifacts, external ventricular drainage affecting hematoma lesions, any other type of surgical intervention that affected ICH volume, or secondary hemorrhage due to acute head trauma, ischemic infarction, neoplastic tumor lesions, ruptured cerebral aneurysms, or vascular malformations. The automated hematoma segmentation and HE classification models were trained using the Yale dataset. The HE classification thresholds were optimized on the ATACH-2 dataset and tested in an external validation dataset from Charité. This study received institutional board approval from all participating centers.

### 2.2. Manual Segmentation of Hematomas on Head CTs as the Ground Truth

The ground truth hematoma lesion masks on admission and follow-up head CT scans were based on manual segmentation by trained research associates at Yale (using 3D-Slicer [22]) and Charité (using ITK-SNAP [16,23]). Hematoma lesions were segmented on axial head CT slices applying 40 to 200 Hounsfield Unit (HU) intensity thresholds to facilitate the distinction of hemorrhages from brain parenchyma, osseous structures, and cerebrospinal fluid. To assess the reliability of segmentations, a subset of scans from both centers underwent additional segmentations to determine intra- and inter-rater reliability using intra-class correlation (ICC).

### 2.3. Automated Hematoma Segmentation Model

- Training framework and dataset: using the Yale cohort dataset, we applied a 5-fold cross-validation framework [24–26] to train and optimize the CNN model for automated segmentation of hematomas on non-contrast head CTs. Then, optimized hyperparameters were used to train the model on the whole Yale dataset;
- Preprocessing of head CT: in the preprocessing step, we removed the skull, adjusted CT scan intensities to the brain window/level, and registered all scans to a common space. For the skull stripping process, we first applied a 0-to-200 HU intensity threshold to remove bony structures followed by a mathematical operation based on head morphology [27] to eliminate any remaining soft tissues using dilation-erosion operations—including binary_erosion(), remove_small_objects(), and binary_dilation()—and removing the boundary with the findContours() function, as described previously [28]. Then, we applied the brain window/level (center = 40 HU, and width = 80 HU), which provides optimal contrast for visualization of brain parenchymal pathologies. Finally, we registered all brain scans to a template, homogenizing the voxel spacing and size (128 × 128 × 128) [28];
- Segmentation model training: we used nnU-Net (no-new-Net) [29] as the backbone for our segmentation model. The nnU-Net (no-new-Net) model is a self-adaptive deep-learning framework designed for medical image segmentation. The model was developed to self-adapt new neural network architecture for each medical image segmentation task, a process that is often time-consuming and requires a grid search strategy. nnU-Net automatically configures itself based on the properties of the provided dataset, adjusting its architecture, pre-processing, and post-processing accordingly. The model has gained considerable popularity in the medical imaging community due to its high performance, ease of use, and proven applicability in a variety of imaging modalities and segmentation tasks. The model was trained in a 5-fold cross-validation process with data augmentation, weight_decay = 3 × 10^−5^, initial_lr = 1 × 10^−2^, num_epochs = 100, PolyLRScheduler, optimizer = SGD, and image input size = (128,128,128);
- Metrics of segmentation performance: we used two metrics to evaluate the hematoma segmentation model performance: the Dice similarity coefficient and the Hausdorff distance. The Dice coefficient [30] measures the volumetric overlap between segmentation results and the ground truth. The Hausdorff distance (HD) [31] measures the surface distance as the maximum distance of a segmentation mask to the nearest point in the ground truth. Details are described in the Supplementary Material.

### 2.4. Optimizing HE Classification Model

**Figure 2.**
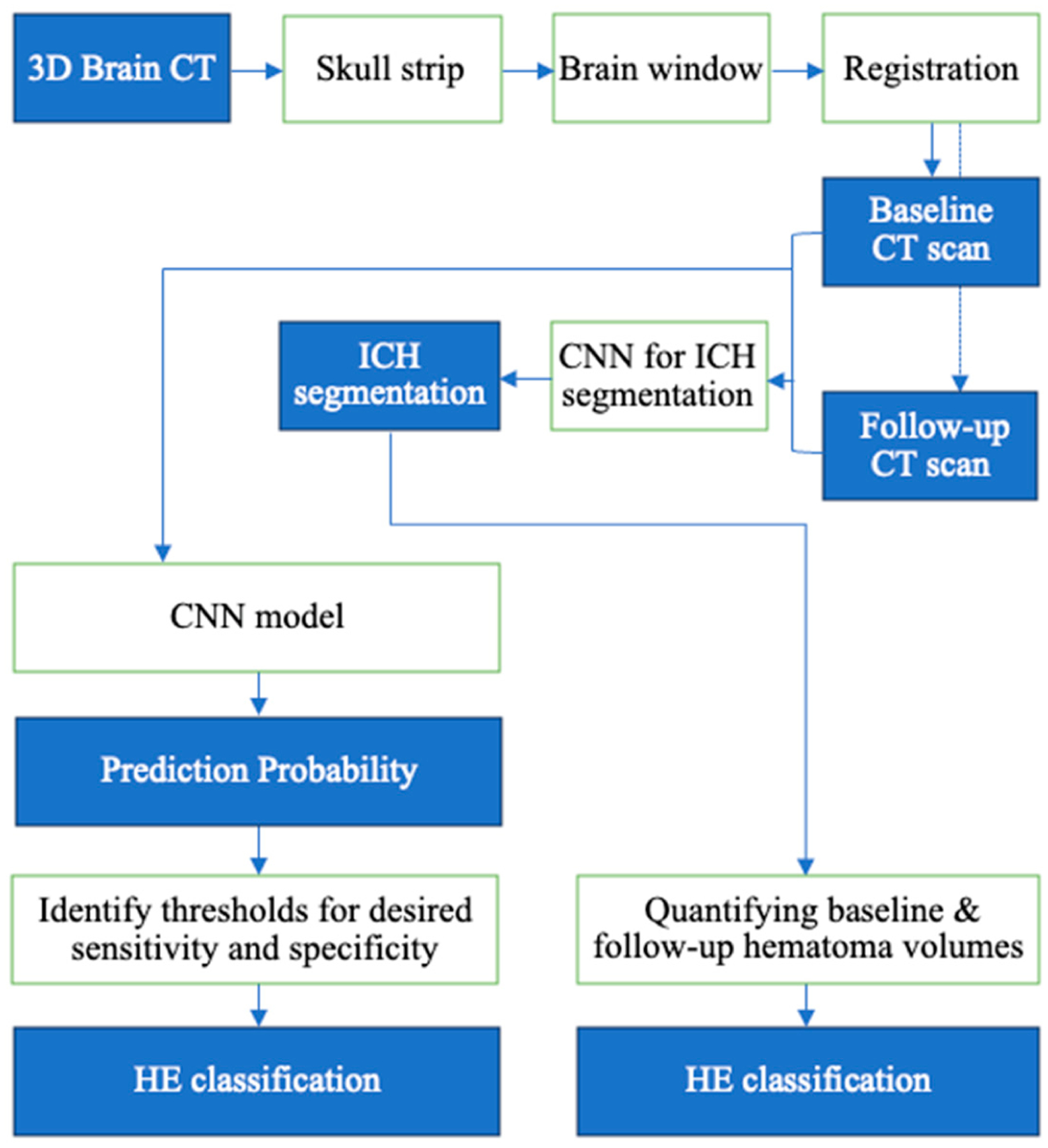
The pipeline for HE classification. Head CT scans were preprocessed for skull stripping, adjusting the intensities to the brain window/level, and resampling and registering to a common size space. The segmentation masks, along with the baseline and follow-up CTs, were used as input for a classification CNN to predict HE. The classifier outputs probability scores for each subject. Then, from the threshold array, sensitivity array, specificity array, and f1 score array, one can choose the optimal threshold; for example, a threshold based on the maximum F1 score [33]. After that, we can create the confusion matrix elements at a given threshold. Using ROC analysis of the final prediction probabilities [34], we established the 100%, 95%, and 90% sensitivity and specificity thresholds in the internal test cohort and evaluated them in the external validation cohort.

- Definition of HE: to facilitate patient risk stratification, HE is typically defined as a binary outcome based on an absolute (e.g., ≥6 mL) or relative (e.g., ≥33%) volume increase. However, since relative volume increases are highly sensitive to potential errors in baseline hematoma quantification and can easily vary for small baseline ICHs, we adopted a series of absolute volume increases for binary definition of HE as proposed previously—i.e., ≥3, ≥6, ≥9, and ≥12.5 mL [13]. Using the manual segmentation of hematomas to generate the baseline (V_baseline_) and follow-up (V_followup_) volumes, the absolute HE was calculated as follows:
$$\text{Absolute}\;\text{HE}=V_{\text{followup}}-V_{\text{baseline}}$$
- Using the *Absolute HE*, we generated binary ground truth classifications for ≥3, ≥6, ≥9, and ≥12.5 mL expansions;
- Direct HE classification based on automated hematoma segmentation results: using volumes from automated hematoma segmentation by the nn-UNET model, we generated a binary HE classification based on absolute volume increases from baseline to follow-up scans;
- Training HE classification models to improve subjects’ annotation (Figure 2): as mentioned in the Introduction section, direct HE classification based on hematoma volumes from automated segmentation is susceptible to cumulative errors from the segmentation model in both baseline and follow-up scans. To address this, we applied CNN models [32] specialized for medical image classification to predict HE annotation with inputs from both admission and follow-up scans as well as segmentation masks. A benefit of such a model is creating a probability range to identify subjects that require further review and potential correction of segmentations based on desired sensitivity or specificity. We applied stratified 5-fold cross-validation, maintaining the ratio of class labels (positive/negative) across each fold. This ensures each fold represents the overall distribution of classes, which is very important when the data are imbalanced. A total of 684 cases from Yale were randomly split (4-to−1) into 548 training cases (136 with HE ≥ 3 mL, 105 with HE ≥ 6 mL, 82 with HE ≥ 9 mL, and 68 with HE ≥ 12.5 mL) and 136 validation cases (35 with HE ≥ 3 mL, 24 with HE ≥ 6 mL, 20 with HE ≥ 9 mL, and 16 with HE ≥ 12.5 mL).
- We modified a DenseNet-121 model [32] to accept four inputs, baseline and follow-up CT scans, along with their respective hematoma segmentation masks, for HE prediction (Figure 2, Figures S1 and S2). The model was trained with loss_function = BCEWithLogitsLoss(), learning rate = 0.0001, weight_decay = 1 × 10^−5^, optimizer = Adam, scheduler = ReduceLROnPlateau, the number of epochs = 100, early stopping, dropout probability = 0.1, and image size = (128,128,128). After cross-validation, the optimized hyperparameters were used to train the final model on the whole dataset.
- Combining CNN with Support Vector Machines (SVMs): the CNN performs two primary tasks: feature extraction using convolution, pooling, and dropout layers, and classification through fully connected and linear layers. Many studies have demonstrated that combining CNNs with SVMs [35] yields better performance than using linear layers in a CNN alone [36,37]. We used the features from the CNN’s final layer as input for an SVM for HE classification. We used C-Support Vector Classification from Sklearn with a Radial Basic Function Kernel and the default configuration [38]. The inputs for the SVM were 4 × 1024 features from the last layer of the DenseNet extracted from baseline and follow-up head CTs and corresponding automated hematoma segmentation masks.

### 2.5. Identifying Subjects for Expert Review of ICH Segmentations, Minimizing HE Misclassification

By transforming the HE classification into prediction probabilities and applying receiver operating characteristic (ROC) analysis, we were able to set sensitivity and specificity thresholds with the goal of limiting expert review of automated hematoma segmentations to subjects at risk of misclassification. A 100% sensitivity threshold ensures no false negatives, limiting expert review to test-positive subjects, whereas a 100% specificity threshold can eliminate false positives, narrowing expert review to test-negative cases (see below formula). This approach optimizes the balance between automated ICH segmentation efficiency and the accuracy of HE classification. However, since achieving 100% sensitivity or specificity may exclude only a small proportion of subjects from hematoma segmentation review, the research team might adopt lower thresholds, allowing a small error rate based on the focus of their study. In our analysis, we established thresholds for 100%, 95%, and 90% sensitivity and specificity using the internal test cohort (ATACH-2) and applied them to the external test cohort (Charité). Additionally, we identified the optimal threshold for maximum accuracy in the internal test and assessed its performance in the external set. For each threshold, we calculated the proportion of subjects requiring expert review of hematoma segmentation due to false-positive or false-negative probabilities.

### 2.6. Statistical Analysis

We applied the statistics and metrics below to determine the classification models’ performance and compare clinical and imaging variables between different cohorts. The details of each metric are further described in the Supplementary Material.

- The confusion matrix [25,39] provides a summary of the prediction results on classification and the true positives (TP), false positives (FP), true negatives (TN), and false negatives (FN) for each class. The metrics obtained from the confusion matrix include accuracy, sensitivity, specificity, and F1 Score [40–42].
- The ROC curve [34] plots the true positive rate (sensitivity/recall) versus the false positive rate (1—specificity) for different threshold values. The AUC (Area Under the Curve) [43] is the area under the ROC curve and provides a measure of the aggregate performance across all classification thresholds. An AUC of 0.5 indicates random prediction, while a value close to 1 indicates strong discrimination;
- The confidence interval (CI) for a performance metric [44] provides a range within which the metric falls with a specified probability (for example, 95% CI);
- Cross-validation (e.g., stratified k-fold cross-validation) splits the data into multiple training and validation sets to obtain a reliable estimate of performance and reduce the risk of overfitting;
- The Chi-Square test [45] is used to examine the relationship between categorical variables or to test the degree of fit between observed data and an expected distribution;
- The independent samples *t*-test [46] is a statistical hypothesis test used to determine whether there is a significant difference between the means of two groups, assuming the data follow a normal distribution,

## 3. Results

### 3.1. Patients’ Characteristics

Table 1 summarizes the demographics, severity of admission symptoms, baseline and follow-up hematoma volumes, the rate of ≥3, ≥6, ≥9, and ≥12.5 mL HE (ground truth), and CT scan characteristics in training/cross-validation (Yale), internal test (ATACH-2), and external validation (Charité) cohorts.

### 3.2. Automated Hematoma Segmentation

The inter- and intra-rater mean ICC of manual hematoma segmentations by the Yale team were 0.92 and 0.94, and by the Charité team they were 0.97 and 0.99, respectively. The nn-UNET-based segmentation model was trained and optimized on the Yale dataset. The automated segmentation model achieved a Dice of 0.86 ± 0.10 and an HD of 3.0 ± 6.9 mm in the internal test (ATACH-2) cohort and a Dice of 0.79 ± 0.19 and an HD of 7.5± 13 mm in the external validation (Charité) cohort. Notably, we found no significant association between hematoma volume and HD in either the ATACH-2 (r = 0.045, *p* = 0.113) or Charité (r = −0.067, *p* = 0.078) cohorts.

### 3.3. HE Classification Directly Based on Automated Segmentation Volumes

Using the hematoma volumes from automated ICH segmentation on baseline and follow-up scans, we achieved an ROC area under curve (AUC) of 0.86 to 0.92 for different HE classifications in the internal test (ATACH-2) cohort and 0.82 to 0.87 in external test (Charité) cohort (Table 2).

### 3.4. HE Classification Based on CNN Classification Model

Table 3 lists the AUC (95% CI) of the CNN classification model using inputs from baseline and follow-up segmentation masks and original head CT scans (Figure 1) versus classification based on automated segmentation volume alone. The AUC of the CNN models predicting ≥3, ≥9, and ≥12.5 mL HE in the ATACH-2 cohort and ≥3 mL HE in the Charité dataset were higher than HE classification using the automated segmentation volumes alone.

**Figure 3.**
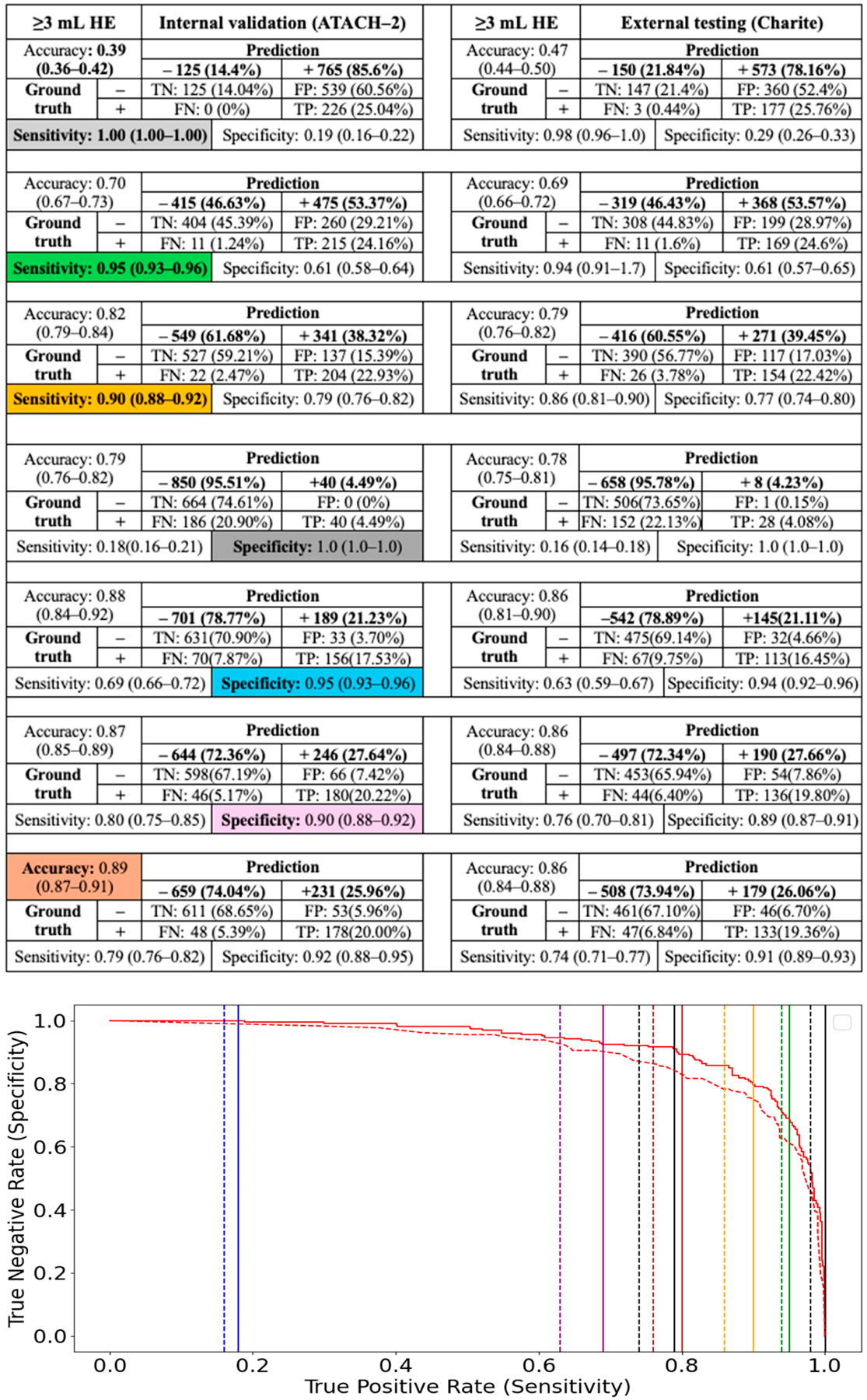
Classification of ≥3 mL HE using CNN model and thresholds for 100%, 95%, and 90% sensitivity and specificity, as well as the highest accuracy threshold, in the internal test cohort (ATACH-2). These thresholds were then applied to the external validation cohort (Charité). The solid and dashed lines in the ROC curve refer to same-color sensitivity/specificity thresholds (as color coded in table cell) in the internal and external validation cohorts, respectively.

**Figure 4.**
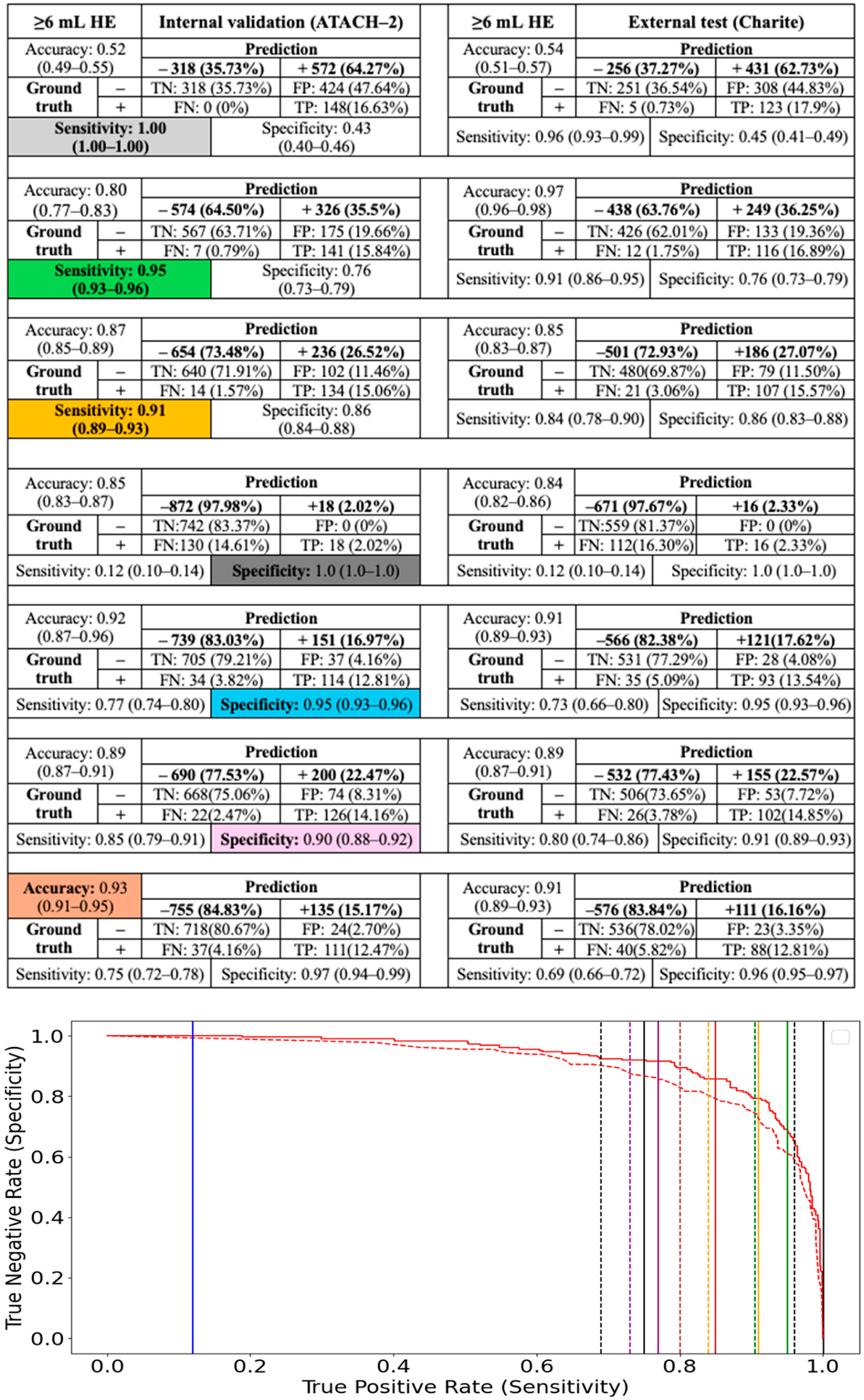
Classification of ≥6 mL HE using CNN model and thresholds for 100%, 95%, and 90% sensitivity and specificity, as well as the highest accuracy threshold, in the internal test cohort (ATACH-2). These thresholds were then applied to the external validation cohort (Charité). The solid and dashed lines in the ROC curve refer to same-color sensitivity/specificity thresholds (as color coded in table cell) in the internal and external validation cohorts, respectively.

**Figure 5.**
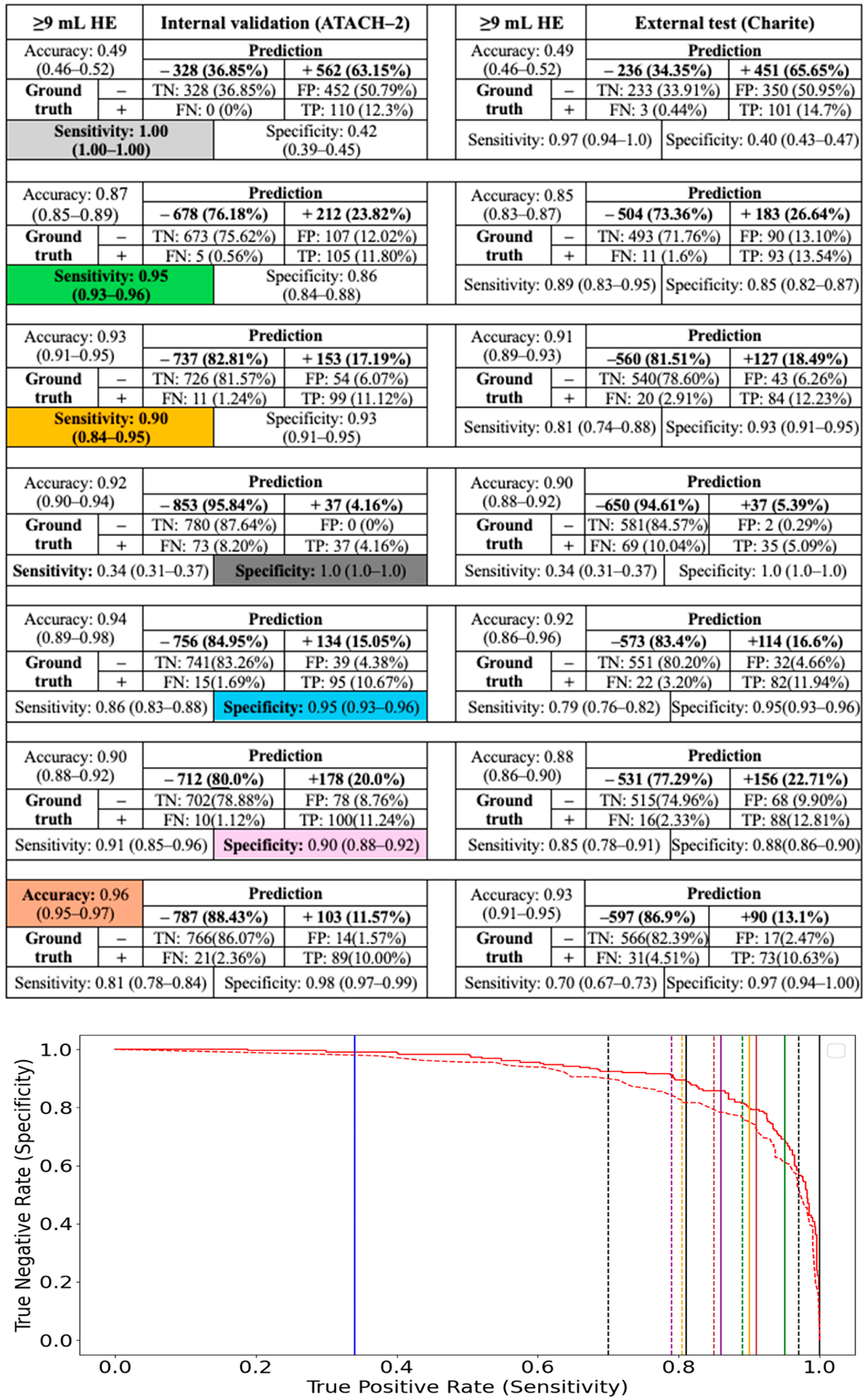
Classification of ≥9 mL HE using CNN model and thresholds for 100%, 95%, and 90% sensitivity and specificity, as well as the highest accuracy threshold, in the internal test cohort (ATACH-2). These thresholds were then applied to the external validation cohort (Charité). The solid and dashed lines in the ROC curve refer to same-color sensitivity/specificity thresholds (as color coded in table cell) in the internal and external validation cohorts, respectively.

**Figure 6.**
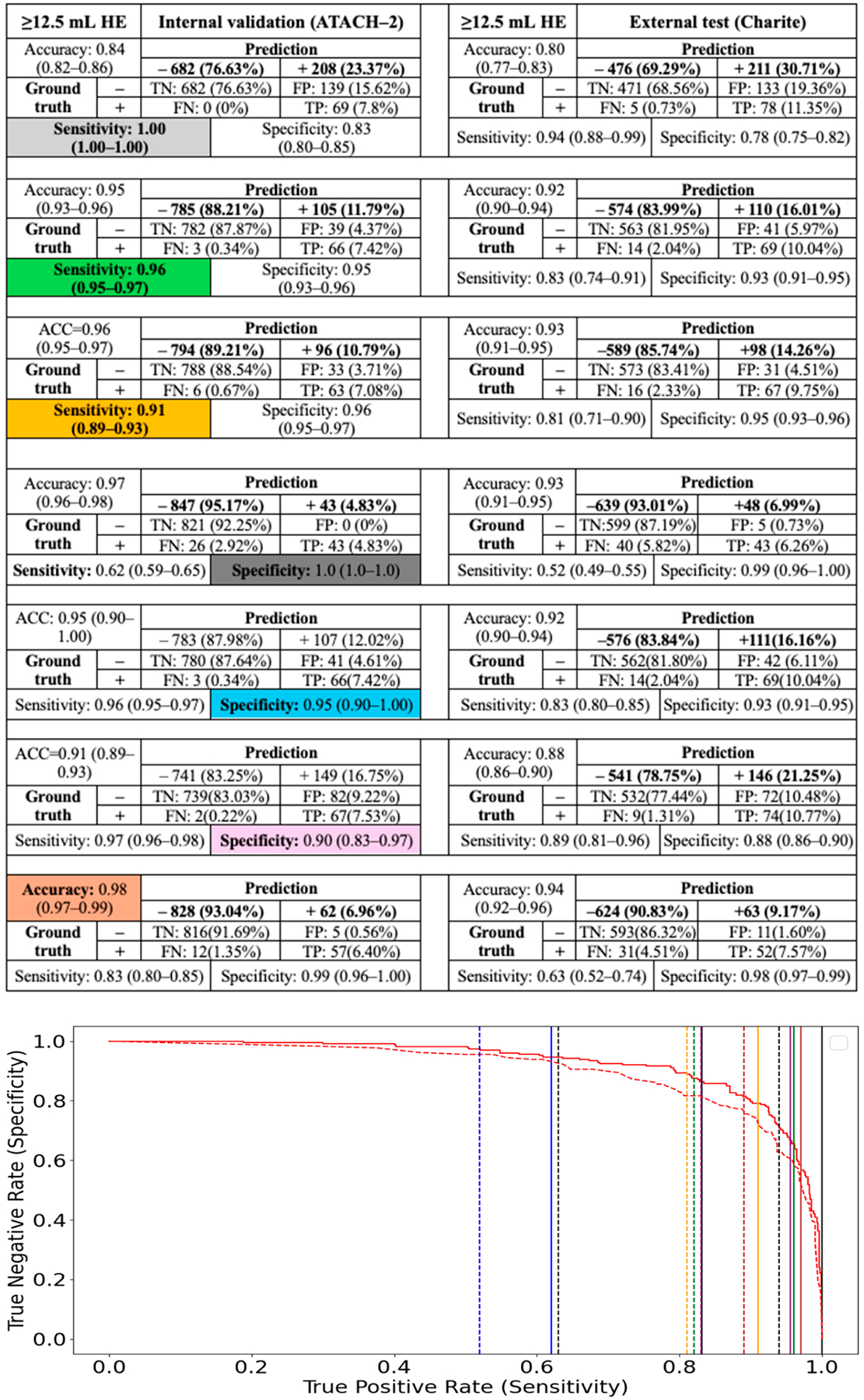
Classification of ≥12.5 mL HE using CNN model and thresholds for 100%, 95%, and 90% sensitivity and specificity, as well as the highest accuracy threshold, in the internal test cohort (ATACH-2). These thresholds were then applied to the external validation cohort (Charité). The solid and dashed lines in the ROC curve refer to same-color sensitivity/specificity thresholds (as color coded in table cell) in the internal and external validation cohorts, respectively.

However, the main advantage of CNN classification models was their ability to establish thresholds that minimize false-negative or false-positive rates, while limiting expert review of segmentations to test-positive or test-negative subjects, respectively. We determined the thresholds for 100%, 95%, and 90% sensitivity and specificity in predicting ≥3, ≥6, ≥9, and ≥12.5 mL HE among the internal test cohort (ATACH-2) and evaluated their performance in the external validation cohort (Charité) (Figures 3–6). High-sensitivity thresholds minimize false-negative rates, thereby limiting expert review of automated segmentations to test-positive subjects. Conversely, high-specificity thresholds minimize false-positive rates, limiting segmentation reviews to test-negative subjects.

Across different HE definitions in the ATACH-2 and Charité cohorts, a 100% sensitivity threshold excluded 14% to 77% of subjects from expert review of automated segmentations (negative prediction), allowing less than 1% false-negative HE misclassification. Conversely, a 100% specificity threshold excluded only 2% to 7% of subjects from expert review of segmentations. High-sensitivity models appear to perform more efficiently in excluding subjects from expert review, while maintaining low false-negative HE misclassification. A more practical threshold might be 95% sensitivity, which could exclude 46% to 88% of subjects from expert review while allowing less than 2% false-negative HE misclassification (Figures 3–6).

### 3.5. HE Classification Based on CNN + SVM Classification Model

Table 4 lists the AUC (95% CI) of the CNN + SVM classification model versus classification based on automated segmentation volume alone. Similar to the CNN model, the AUC of the CNN + SVM models predicting ≥3, ≥9, and ≥12.5 mL HE in the ATACH-2 cohort and ≥3 mL HE in the Charité dataset were higher than HE classification using the automated segmentation volumes alone.

For CNN + SVM models, we also determined the thresholds for 100%, 95%, and 90% sensitivity and specificity in predicting ≥3, ≥6, ≥9, and ≥12.5 mL HE among the internal test cohort (ATACH-2) and evaluated their performance in the external validation cohort (Charité) (Supplemental Figures S3–S7). The CNN + SVM model reduced the false-negative misclassification rate in Charité external validation (Table 5).

Although 100% sensitivity and specificity thresholds for the CNN + SVM models maintained their performance in the Charité external validation cohort, they could exclude fewer than 10% of subjects from expert review of automated segmentation (Supplemental Figures S4–S7). On the other hand, 95% and 90% sensitivity thresholds maintained their performance in the external validation cohort with a 1 to 3% false-negative rate in HE classification. For example, applying the 90% sensitivity threshold can exclude 35–90% of test-negative predictions from expert review of automated segmentations, allowing for a 0.7–2.8% false-negative rate. Similar to the CNN model, high-specificity thresholds for the CNN + SVM models were less effective in minimizing false-positive rates than high-sensitivity thresholds were for reducing false-negative rates.

### 3.6. Model Execution

We ran the final segmentation model on a system using Python in Ubuntu 22, with a CPU AMD Ryzen 3975WX and a Quadro RTX 6000 GPU with 24 GB of memory. The test was completed in 20 ± 3 s including pre-processing, segmentation, and classification.

## 4. Discussion

Large stroke datasets and machine-learning models offer a unique opportunity to facilitate decision-making in acutely critical settings, optimize personalized acute interventions, and tailor post-discharge rehabilitation plans to individual needs [47,48]. As a modifiable risk factor and a potential treatment target in acute ICH, HE has been a focal point of research over the past few decades [9,10,49–51]. Recently, some studies have also developed deep learning models to predict HE from admission head CT scans, with reported AUC values in the 0.8 range [28,52–56]. However, the very first step in evaluating HE is the creation of ground truth data, whether the objective is to study HE as a risk factor or to train and test predictive deep learning models. To date, all prior studies have relied on either manual segmentation of hematomas on admission and follow-up head CT scans or semi-automated segmentation followed by manual revisions [9,10,28,49–56]. Both approaches are time-consuming and labor-intensive. In this study, we proposed a novel pipeline for generating ground truth data for HE. This pipeline incorporates automated segmentation of hematomas on admission and follow-up scans, followed by a classification model with an optimized threshold to minimize classification errors. Our approach generates HE class annotations from admission and follow-up CT scans and identifies a subset of subjects that require visual review and manual correction of automated segmentation, thereby reducing cumulative errors from the automated segmentation model in baseline and follow-up head CTs. The pipeline generated accurate HE labels for over 98% of subjects in both the internal and external validation cohorts, while reducing the need for expert review to 10%–50% of subjects, depending on the HE definition.

Although automated segmentation of hematomas is completed within seconds, reviewing the results to ensure accuracy can be time-consuming. On average, manual segmentation of hematomas takes 10 to 30 min per subject [15,57,58]. Automated segmentation followed by manual review or semi-automated segmentation reduces this time to 3 to 5 min per case [15,57,58]. Even a simple review of segmentation results without further corrections requires 1 to 3 min per case. Our pipeline optimizes the process of annotating patients with HE as the ground truth by limiting expert review of segmentation to approximately one-third of the dataset. For a modest dataset of about 300 patients, this approach can save over 7 h of expert review time.

Many groups have reported the application of deep learning models for automated segmentation of ICH [14–20,57–59] or the prediction of HE from admission head CT scans [28,52–56]. However, no study to date has applied a combined segmentation and classification model to generate ground truth labels of HE in ICH datasets. Some groups have utilized image synthesis to improve ICH detection and classification on CT scans [60,61], whereas our goal was to mitigate the cumulative errors in hematoma volume measurements from automated segmentation across two separate head CT scans, at admission and follow-up, when calculating the absolute volume increases for binary HE classification. The primary application of our pipeline is to reduce the time required for expert visual review and correction of automated hematoma segmentations in large ICH datasets.

From a practical standpoint, although 100% sensitivity and specificity thresholds for HE classification maintained their performance in the external validation cohort, they were able to exclude only a small proportion of subjects from expert review of hematoma segmentation. Therefore, it may be more practical to apply high (e.g., 90% or 95%) sensitivity or specificity thresholds, allowing for a small percentage of false-negative or false-positive misclassifications, depending on which type of misclassification is permissible for given research scenarios. Overall, we demonstrated that for HE classification, high-sensitivity thresholds were more effective at minimizing false-negative rates while excluding a larger proportion of test-negative subjects from expert review, compared to high-specificity thresholds, which resulted in a higher false-positive rate and excluded a smaller proportion of (test-positive) subjects from automated segmentation review.

From a clinical standpoint, high-sensitivity thresholds are crucial to ensure the model identifies the majority of patients with HE, thereby minimizing the risk of missing cases with expansion. In practice, there is often a preference to over-diagnose rather than under-diagnose individuals at risk of life-threatening conditions such as HE. High-sensitivity models are also well-suited to serve as components within larger, multimodal risk stratification systems. While our primary objective was to generate ground truth HE labels for a large ICH dataset, rather than to identify high-risk individuals from an admission head CT, accurately identifying and labeling patients with HE remains essential. Figure 1 illustrates how our workflow achieved > 99% accurate classification across the entire cohort, with >95% of HE cases correctly labeled. Importantly, expert reviewers only need to review and correct segmentations in approximately one-third of the dataset, significantly improving efficiency while maintaining accuracy.

The combination of CNN and SVM has many advantages [62–64]. The CNN classification models are specialized for image feature extraction and learn hierarchical features, capturing both low-level and high-level patterns from images. SVMs are specialized to maximize the margin between classes, which often leads to more effective and sharper decision boundaries compared to softmax-based approaches in the final layer of CNN classification models [65,66]. SVMs are also well-suited for handling high-dimensional feature spaces and can offer better generalization as a classifier on top of a CNN’s feature extraction layers, potentially improving the model’s robustness to overfitting [36,37].

The main advantage of using a CNN + SVM over a CNN model was maintaining sensitivity consistency across training and testing datasets. A CNN classifier directly outputs probabilities via a 4-sigmoid activation, which is trained to optimize by loss functions. A sensitivity threshold is applied to these probabilities. However, CNN predictions are closely tied to the training datasets, and imbalanced datasets can skew the probability distribution, leading to suboptimal threshold generalization. The CNN in our approach acts as a feature extractor, and the extracted features are fed into an SVM classifier. The SVM learns decision boundaries in the feature space instead of predicting probabilities directly. Probabilities are inferred later, but SVM decision boundaries are typically more robust to imbalanced data, improving generalization across datasets. SVM-based probabilities are typically more heavily calibrated than the raw sigmoid output of the CNN, leading to better threshold selection across datasets. Since the probability distribution of a CNN is not consistent across datasets, the CNN + SVM approach translates the features extracted from the CNN into robust decision boundaries, making it less susceptible to dataset-specific variations in threshold behavior.

In our study, we adopted absolute volume increase thresholds for the definition of HE. Using relative rather than absolute volume increases to define HE can introduce marked variability due to its dependence on the initial hematoma volume. For instance, in the case of a small initial hematoma volume, such as 1 mL, a 33% increase—proposed by some as a threshold for binary classification [13]—results in a cutoff volume of 1.33 mL. This means that a follow-up hematoma measuring 1.4 mL would be classified as having expanded, while a hematoma measuring 1.3 mL would not, despite the absolute difference being only 0.1 mL. This small difference falls within the range of typical segmentation or measurement variability, making the classification highly sensitive to minor inaccuracies. Additionally, the reliance on a percentage increase disproportionately penalizes smaller hematomas, where slight volumetric changes represent a higher relative change, compared to larger hematomas where substantial volume increases might still fall below the threshold. This creates a systematic bias and potential misclassification, highlighting the limitations of using relative volume thresholds to define HE in a consistent and clinically meaningful manner.

Segmentation of small hematomas can be particularly challenging for deep learning models due to their size and limited features. To address this, some research groups have implemented model design modifications, such as a multi-scale learning-to-rank framework within a U-Net architecture [59], to improve the segmentation of tiny hematomas [8]. In our study, however, we found no significant association between hematoma volume and segmentation Dice scores, indicating no systematic errors related to hematoma size. Furthermore, there were no instances of HE misclassification for baseline hematomas with volumes <1 mL. This outcome is likely due to our use of clinically relevant absolute HE definitions, starting at a threshold of ≥3 mL, which minimizes the influence of inaccuracies in segmenting very small hematomas.

Differences in patient population characteristics, scanning parameters, and inter-reviewer variability in hematoma segmentation can potentially hamper the trans-institutional generalizability of our pipeline. However, our use of the multicentric ATACH-2 cohort for internal testing and for establishing sensitivity and specificity thresholds, combined with the validation of the final pipeline on an unseen external cohort from Charité—where hematomas on head CTs were delineated by an independent research team—suggest that our pipeline and thresholds are likely to be generalizable.

Our results were limited by a sizable proportion of subjects that may need expert review of automated segmentations. The differences in cross-validation/training, internal test, and external validation cohorts might have contributed to maintaining sensitivity and specificity thresholds. The datasets used for external validation were obtained from ICH patients who presented to the respective centers prior to 2019. This timeline accounts for the inevitable delays associated with data collection, quality control, image processing, and hematoma segmentation. Finally, it should be noted that even human segmentations are inevitably prone to error and inter-reviewer variability.

## 5. Conclusions

We developed and tested a hybrid model combining hematoma segmentation and HE classification to generate binary absolute HE annotations in a large ICH dataset. This approach uses automated hematoma segmentation on admission and follow-up head CTs, followed by a deep-learning HE class prediction model, to limit expert review of automated segmentation to a subset of patients based on preferences to minimize false-positive or false-negative misclassifications in the final classification. Further improvements in segmentation and classification models can increase the proportion of subjects excluded from expert review of segmentations.

## Supplementary Material

Supplementary Material

## Figures and Tables

**Table 1.** Comparison of patients’ characteristics, hematoma volumes, and CT scan variables in different study cohorts.

	Yale * (*n* = 684)	ATACH-2 * (*n* = 890)	Charité * (*n* = 687)	*p* Value
Male	358 (54.8%)	543 (60.80%)	530 (56,36%)	0.345
Age [years]	69.7 ± 14.3	62.2 ± 13.1	69.4 ± 14.2	<0.001
Baseline NIH Stroke Scale	8 (2 −17)	11 (6–16)	6 (1–12)	<0.001
Baseline ICH volume [mL]	18.7 ± 20.6	12.9 ± 12.5	30.2 ± 30.7	<0.001
Follow-up ICH volume [mL]	22.9 ± 25.8	15.6 ± 16.6	31.9 ± 34.5	<0.001
≥3 mL HE	171 (25%)	226 (25.4%)	180 (26.2%)	0.872
≥6 mL HE	129 (18.9%)	148 (16.6%)	128 (18,6%)	0437
≥9 mL HE	102 (14.9%)	110 (12.4%)	104 (15.1%)	0.201
≥12.5 mL HE	84 (12.3%)	69 (7.7%)	83 (12.1%)	0.006
CT scan characteristics				
In-plane pixel spacing [mm]	[0.46 × 0.46]	[0.46 × 0.46]	[0.46 × 0.46]	
Slice thickness [mm]	4.81 ± 0.70	5.29 ± 1.81	4.54 ± 0.66	<0.001
Min axial image matrix [*n* × *n*]	[472 × 472]	[512 × 512]	[434 × 434]	
Max axial matrix [*n* × *n*]	[1024 × 1024]	[512 × 734]	[512 × 671]	
Number of slices	35.9 ± 11.6	30.9 ± 17.6	30.26 ± 7.8	<0.001

* Yale dataset was used for training/cross-validation of hematoma segmentation and HE prediction models; ATACH-2 dataset was used as an internal test to determine thresholds for different sensitivity and specificity predictions; and Charité dataset was used as an external test cohort.

**Table 2.** HE classification based on hematoma volumes from automated ICH segmentation model.

	Internal Independent Test (ATACH-2, *n* = 890)				External Independent Test (Charité, *n* = 687)			
	AUC	Accuracy	Sensitivity	Specificity	AUC	Accuracy	Sensitivity	Specificity
**≥3 mL HE**	0.86 (0.84–0.88)	0.90 (0.88–0.92)	0.76 (0.70–0.81)	0.95 (0.93–0.97)	0.82 (0.79–0.85)	0.87 (0.84–0.90)	0.71 (0.64–0.78)	0.93 (0.91–0.95)
**≥6 mL HE**	0.89 (0.87–0.91)	0.95 (0.93–0.96)	0.82 (0.76–0.88)	0.97 (0.96–0.98)	0.88 (0.86–0.90)	0.94 (0.92–0.96)	0.79 (0.72–0.86)	0.97 (0.96–0.98)
**≥9 mL HE**	0.89 (0.87–0.91)	0.96 (0.95–0.97)	0.80 (0.73–0.87)	0.98 (0.97–0.99)	0.88 (0.86–0.90)	0.95 (0.93–0.97)	0.78 (0.70–0.86)	0.98 (0.97–0.99)
**≥12.5 mL HE**	0.92 (0.90–0.94)	0.98 (0.97–0.99)	0.86 (0.77–0.94)	0.99 (0.98–1.00)	0.88 (0.85–0.99)	0.95 (0.93–0.97)	0.76 (0.66–0.84)	0.98 (0.97–0.99)

The values are presented with 95% CI.

**Table 3.** Comparison of HE classification based on CNN classification model versus direct subtraction of hematoma volumes from automated segmentation.

	Internal Independent Test (ATACH-2, *n* = 890)			External Independent Test (Charité, *n* = 687)		
	Classification by CNN Model	Classification Based on Segmentation Volumes	*p* Value	Classification by CNN Model	Classification Based on Segmentation Volumes	*p* Value
**≥3 mL HE**	0.93 (0.90–0.95)	0.86 (0.84–0.88)	0.005	0.9 (0.87–0.92)	0.82 (0.79–0.85)	0.018
**≥6 mL HE**	0.95 (0.94–0.97)	0.89 (0.87–0.91)	0.175	0.93 (0.90–0.95)	0.88 (0.86–0.90)	0.520
**≥9 mL HE**	0.97 (0.95–0.98)	0.89 (0.87–0.91)	0.022	0.93 (0.91–0.95)	0.88 (0.86–0.92)	0.525
**≥12.5 mL HE**	0.98 (0.98–0.99)	0.92 (0.90–0.94)	0.037	0.95 (0.93–0.98)	0.87 (0.85–0.99)	0.882

The ROC AUC (95% CI) of HE classification based on CNN classification versus directly from automated segmentation volumes.

**Table 4.** Comparison of HE classification based on CNN + SVM classification model versus direct subtraction of hematoma volumes from automated segmentation.

	Internal Independent Test (ATACH-2, *n* = 890)			External Independent Test (Charité, *n* = 687)		
	Prediction Model	Segmentation Volumes	*p* Value	Prediction Model	Segmentation Volumes	*p* Value
**≥3 mL HE**	0.91 (0.89–0.93)	0.86 (0.83–0.88)	0.006	0.88 (0.84–0.91)	0.82 (0.78–0.85)	0.019
**≥6 mL HE**	0.92 (0.89–0.95)	0.90 (0.86–0.92)	0.286	0.9 (0.86–0.94)	0.88 (0.85–0.92)	0.529
**≥9 mL HE**	0.94 (0.92–0.97)	0.89 (0.85–0.93)	0.033	0.9 (0.85–0.94)	0.88 (0.84–0.92)	0.552
**≥12.5 mL HE**	0.98 (0.95–1)	0.92 (0.88–0.96)	0.038	0.87 (0.82–0.93)	0.87 (0.82–09.3)	0.928

The ROC AUC (95% CI) of HE classification based on CNN + SVM classification versus directly from automated segmentation volumes.

**Table 5.** Comparison of false-negative rates from CNN and CNN + SVM classification models as well as direct subtraction of hematoma volumes from segmentation model.

False-Negative Misclassification Rate with Each Model		100% Sensitivity		95% Sensitivity		90% Sensitivity		Direct from Segmentation
		CNN Only	CNN/SVM	CNN Only	CNN/SVM	CNN Only	CNN/SVM	
**ATACH-2**	**≥3 mL HE**	0 (0.0%)	0 (0.0%)	11 (1.2%)	11 (1.2%)	22 (2.5%)	22 (2.5%)	53(5.9%)
	**≥6 mL HE**	0 (0.0%)	0 (0.0%)	7 (0.7%)	7 (0.7%)	14 (1.6%)	14 (1.6%)	23(2.6%)
	**≥9 mL HE**	0 (0.0%)	0 (0.0%)	5 (0.5%)	5 (0.5%)	11 (1.2%)	11 (1.2%)	19(2.1%)
	**≥12.5 mL HE**	0 (0.0%)	0 (0.0%)	3 (0.3%)	3 (0.3%)	6 (0.6%)	6 (0.6%)	9(1.0%)
**Charité**	**≥3 mL HE**	3 (0.4%)	0 (0.0%)	11 (1.6%)	5 (0.7%)	26 (3.8%)	14 (2.0%)	46(6.7%)
	**≥6 mL HE**	5 (0.7%)	0 (0.0%)	12 (1.7%)	7 (1.0%)	21 (3.1%)	12 (1.7%)	22(3.2%)
	**≥9 mL HE**	3 (0.4%)	0 (0.0%)	11 (1.6%)	2 (0.3%)	20 (2.9%)	14 (2.0%)	21(3.1%)
	**≥12.5 mL HE**	5 (0.7%)	2 (0.3%)	14 (2.0%)	16 (2.3%)	16 (2.3%)	19 (2.7%)	17(2.5%)

## Data Availability

Data are available upon request and on approval from the respective register holders.
