# Supplementary material for: Optimizing Automated Hematoma Expansion Classification from Baseline and Follow-Up Head Computed Tomography
Source: Appl Sci (Basel). Author manuscript; Available in PMC 2025 Mar 5. (PMC11882137; doi:10.3390/app15010111)
Supplement: Supplementary Material [file NIHMS2052981-supplement-Supplementary_Material.pdf]

Supplementary Material

1 Evaluation of model performance 2
2 Figure S1. CNN architecture 4
3 Figure S2. Example loss and AUC diagram when training/validation 5
4 Figure S3. Histogram of HE annotation from model (CNN+SVM) with high sensitivity=0.90 ...6
5 Figure S4. Classification of ≥3 mL HE using CNN+SVM model...7
6 Figure S5. Classification of ≥6 mL HE using CNN+SVM model...8
7 Figure S6. Classification of ≥9 mL HE using CNN+SVM model...9
8 Figure S7. Classification of ≥12.5 mL HE using CNN+SVM model......................................10

1 Evaluation of model performance

- *Segmentation:*

Dice Similarity Coefficient [1] measures the volumetric overlap between segmentation results and the ground truth, with values ranging from 0 to 1, where 1 represents a perfect match. The Dice score quantifies the degree of overlap between the model's segmentation and the ground truth masks, with higher values indicating greater segmentation accuracy. By considering both the intersection and the total number of elements in each set, the Dice coefficient provides a balance between sensitivity (true

positive rate) and positive predictive value (precision).

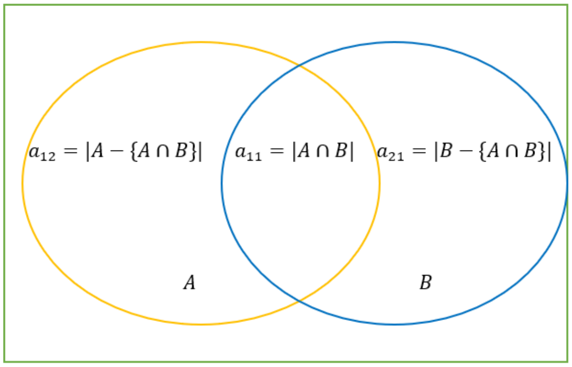

$$\boldsymbol{Dice} = \frac{\boldsymbol{2a_{11}}}{\boldsymbol{2a_{11} + a_{12} + a_{21}}}$$

Hausdorff distance (HD) [2] which measures surface distance is the maximum distance of a set to the nearest point in the other set defined as

$$d_H(X, Y) = \max\{d_{XY}, d_{YX}\} = \max\left\{\max_{x \in X} \min_{y \in Y} d(x, y), \max_{y \in Y} \min_{x \in X} d(x, y),\right\} \quad (2)$$

Given two sets of points, A and B,

- For every point a in A, find the minimum distance to any point in B, and then take the maximum of these minimum distances (this is the directed distance from A to B).
- Repeat the process in the other direction (from B to A).
- The Hausdorff Distance is the greater of these two directed distances.

Below is an example of Hausdorff Distance (the greater of these two directed distances).

$HD \in [0, \sqrt{W^2 + H^2 + D^2}]$ in 3D images. Hausdorff Distance is the greater

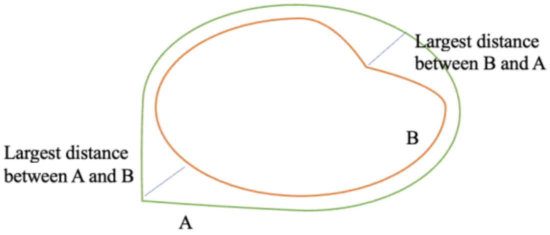

- *classification*

The confusion matrix [3,4] provides a summary of the prediction results on a classification, the true positives (TP), false positives (FP), true negatives (TN), and false negatives (FN) for each class. True versus false represent the ground truth status, and positive versus negative refers to classification prediction. The metrics obtained from the confusion matrix include Accuracy, Sensitivity, Specificity, and F1 Score.

- $Accuracy = \frac{TP+TN}{TP+TN+FP+FN}$
- $Sensitivity = \frac{TP}{TP+FN}$
- $Specificity = \frac{TN}{TN+FP}$
- $Precision = \frac{TP}{TP+FP}$
- $F1-score = \frac{2*Precision*Sensitivity}{Precision+Sensitivity}$

The receiver operating characteristics (ROC) curve [5] plots the true positive rate (sensitivity/recall) versus the false positive rate (1 - specificity) for different threshold values. The area under the curve (AUC) of ROC provides a measure of the aggregate performance across all classification thresholds [6]. An AUC of 0.5 indicates random prediction, while a value of 1 indicates perfect discrimination.

Confidence interval (CI) for a performance metric provides a range within which the metric falls with a specified probability (e.g., 95% CI).

$$CI = \hat{p} \pm z * \sqrt{\frac{\hat{p} * (1-\hat{p})}{N}}$$

Where $\hat{p}$ sample accuracy, z: Z-score, N: Total number of samples

Cross-validation (e.g., stratified k-fold cross-validation) [7] splits the data into multiple training and validation sets to obtain a reliable estimate of performance and reduce the risk of overfitting.

The Chi-Square test [8] is a statistical test used to test the relationship between categorical variables or to test the degree of fit between observed data and an expected distribution.

$$\mathrm{X}^2 = \sum \frac{(O-E)^2}{E}$$

Where O = Observed frequency, E = Expected frequency

The t-test [9] is a statistical hypothesis test used to determine whether there is a significant difference between the means of two groups, assuming the data follows a normal distribution.

$$t = \frac{\overline{x_1} - \overline{x_2}}{\sqrt{\frac{{s_1}^2}{n_1} + \frac{{s_2}^2}{n_2}}}$$

where: $\overline{x_1}, \overline{x_2}$: Sample means; $s_1,\ s_2$: Standard deviations; $n_1,\ n_2$: Sample sizes

The null hypothesis (H_0) was that machine learning models cannot classify patients with and without hematoma expansion from baseline and follow-up head CTs, and alternative hypothesis (H_1) was that machine learning models can classify patients with and without hematoma expansion from baseline and follow-up head CTs.

2 Figure S1. CNN architecture

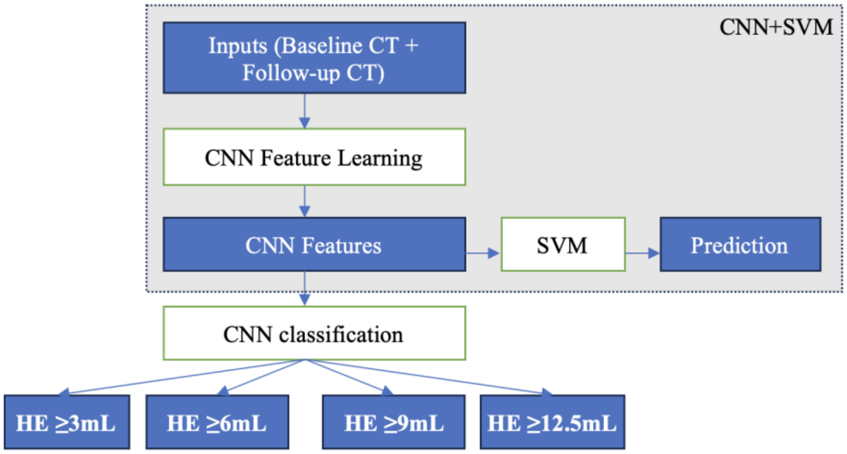


```
class MyDenseNet(nn.Module):
........................
    def forward(self, input: torch.Tensor) -> torch.Tensor:
                x = self.features(input)
                features1 = self.class_layers(x)        #1024
                features2 = self.class_layers(x)        #1024
                features3 = self.class_layers(x)        #1024
                features4 = self.class_layers(x)        #1024
                x1 = self.fc(features1)                 #1
                x2 = self.fc(features2)                 #1
                x3 = self.fc(features3)                 #1
                x4 = self.fc(features4)                 #1
                return x1, x2, x3, x4, features1, features2, features3, features4
```

https://github.com/anhtrnyaleedu/HEAnnotation/blob/main/model.architecture.png
https://github.com/anhtrnyaleedu/HEAnnotation/blob/main/MyDenseNet121.py

3 Figure S2. Example loss and AUC diagram when training/validation

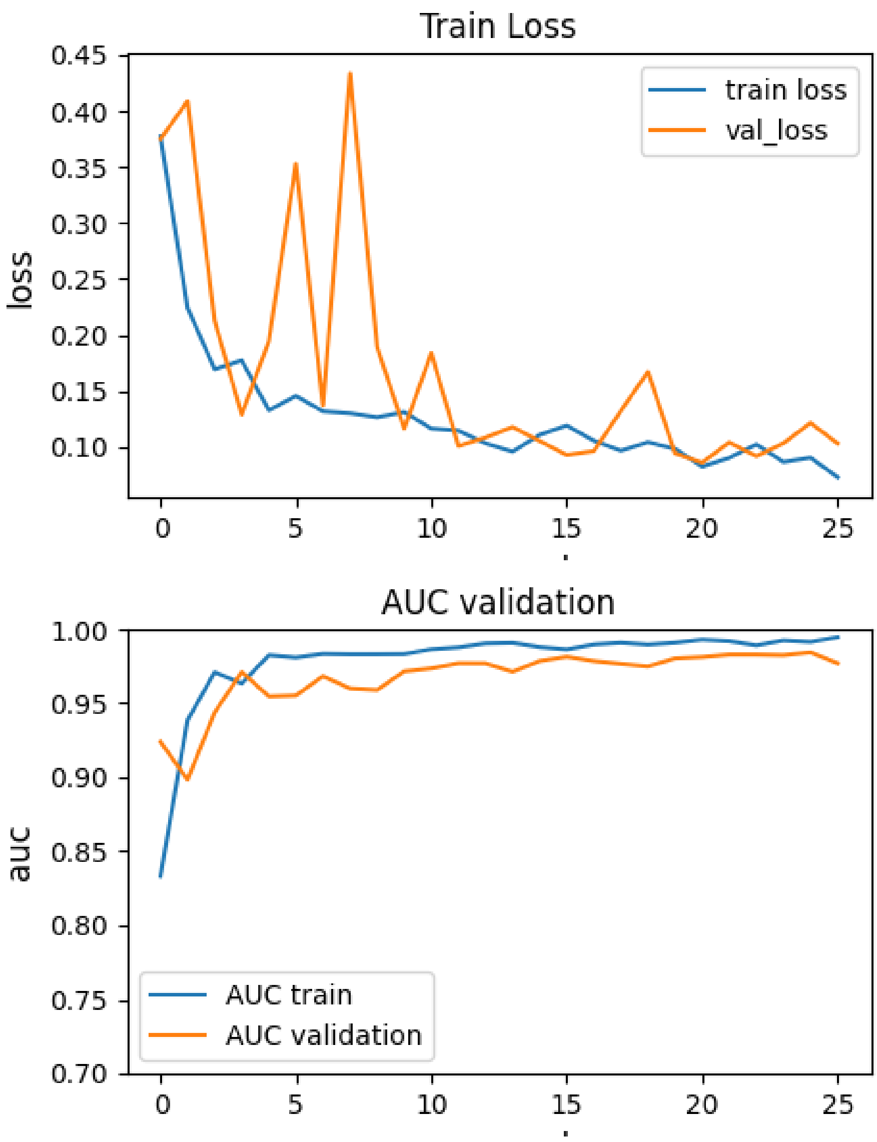

4 Figure S3. Histogram of HE annotation from model (CNN+SVM) with high sensitivity=0.90

- **ATACH-2 test cohort**

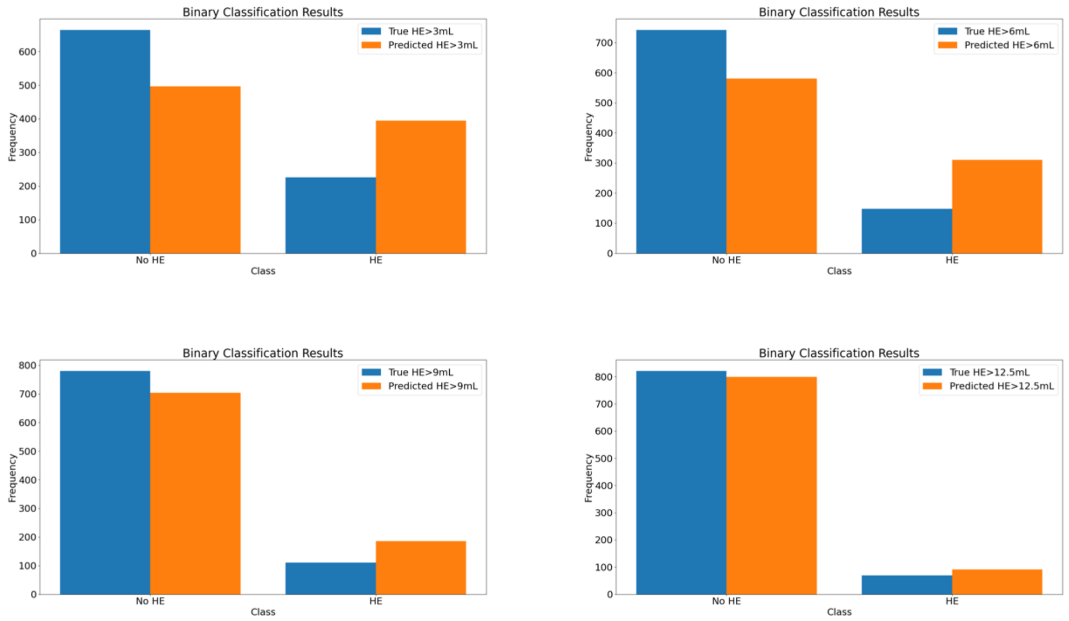

- **Charité test cohort**

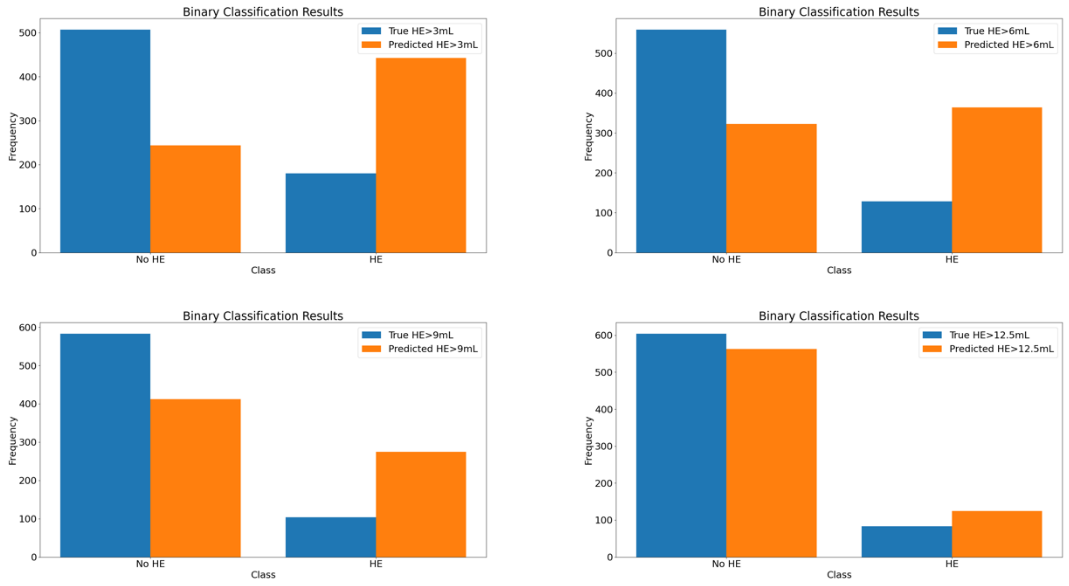

Figure S4. Classification of ≥3 mL HE using CNN+SVM model

≥3 mL HE		Internal test (ATACH-2)		≥3 mL HE		External validation (Charite)	
**Accuracy: 34%**		**Prediction**		Accuracy: 28%		**Prediction**	
		**– 75 (8.4%)**	**+ 815 (91.6%)**			**- 14 (2%)**	**+ 573 (88%)**
**Ground truth**	–	TN: 75 (8.4%)	FP: 589 (66.2%)	**Ground truth**	–	TN: 14 (2%)	FP: 493 (71.7%)
	+	FN: 0 (0%)	TP: 226 (25.4%)		+	FN: 0 (0%)	TP: 180 (26.3%)
**Sensitivity: 100%**		Specificity:11%		Sensitivity: 100%		Specificity: 3%	
Accuracy: 63%		**Prediction**		Accuracy: 46%		**Prediction**	
		**– 354 (40%)**	**+ 536 (60%)**			**– 148 (21.5%)**	**+ 539 (78.5%)**
**Ground truth**	–	TN: 343 (39%)	FP: 321 (36%)	**Ground truth**	–	TN: 143 (20.8%)	FP: 364 (53%)
	+	FN: 11 (1%)	TP: 215 (24%)		+	FN: 5 (0.7%)	TP: 175 (25.5%)
**Sensitivity: 95%**		Specificity: 52%		Sensitivity: 97%		Specificity: 28%	
Accuracy: 76%		**Prediction**		Accuracy: 58%		**Prediction**	
		**– 496 (55.7%)**	**+ 394 (44.3%)**			**– 244 (35.5%)**	**+ 443 (64.5%)**
**Ground truth**	–	TN: 474 (53.2%)	FP: 190 (21.3%)	**Ground truth**	–	TN: 230 (33.5%)	FP: 277 (40.3%)
	+	FN: 22 (2.5%)	TP: 204 (23%)		+	FN: 14 (2%)	TP: 166 (24.2%)
**Sensitivity: 90%**		Specificity: 71%		Sensitivity: 92%		Specificity: 45%	
Accuracy: 78%		**Prediction**		Accuracy: 74%		**Prediction**	
		**– 677 (96.4%)**	**+32 (3.6%)**			**– 679 (98.9%)**	**+ 8 (1.1%)**
**Ground truth**	–	TN: 664 (74.6%)	FP: 0 (0%)	**Ground truth**	–	TN: 504 (73.4%)	FP: 3 (0.4%)
	+	FN:194 (21.8%)	TP: 32 (3.6%)		+	FN:175 (25.5%)	TP: 5 (0.7%)
Sensitivity:14%		**Specificity:100%**		Sensitivity: 3%		Specificity: 99%	
Accuracy: 88%		**Prediction**		Accuracy: 96%		**Prediction**	
		**– 704 (79.1%)**	**+ 186 (20.9%)**			**-526 (76.6%)**	**+161(23.4%)**
**Ground truth**	–	TN: 631 (70.9%)	FP: 33 (3.7%)	**Ground truth**	–	TN: 467 (68%)	FP: 40 (5.8%)
	+	FN: 73 (8.2%)	TP: 153 (17.2%)		+	FN: 59 (8.6%)	TP: 121 (17.6%)
Sensitivity: 68%		**Specificity: 95%**		Sensitivity: 67%		Specificity: 92%	
Accuracy: 87%		**Prediction**		Accuracy: 81%		**Prediction**	
		**– 652 (73.3%)**	**+ 238 (26.7%)**			**- 460 (67%)**	**+ 227 (33%)**
**Ground truth**	–	TN: 598 (67.2%)	FP: 66 (7.4%)	**Ground truth**	–	TN: 419 (61%)	FP: 88 (12.8%)
	+	FN: 54 (6.1%)	TP: 172 (19.3%)		+	FN: 41 (6%)	TP: 139 (20.2%)
Sensitivity: 76%		**Specificity: 90%**		Sensitivity: 77%		Specificity: 83%	
**Accuracy: 88%**		**Prediction**		Accuracy: 84%		**Prediction**	
		**– 677 (76%)**	**+213 (24%)**			**– 493 (71.7%)**	**+ 194 (28.3%)**
**Ground truth**	–	TN: 618 (69.4%)	FP: 46 (5.2%)	**Ground truth**	–	TN: 444 (64.6%)	FP: 63 (9.2%)
	+	FN:59 (6.6%)	TP: 167 (18.8%)		+	FN:49 (7.1%)	TP: 131 (19.1%)
Sensitivity:74%		Specificity:93%		Sensitivity: 73%		Specificity: 88%	

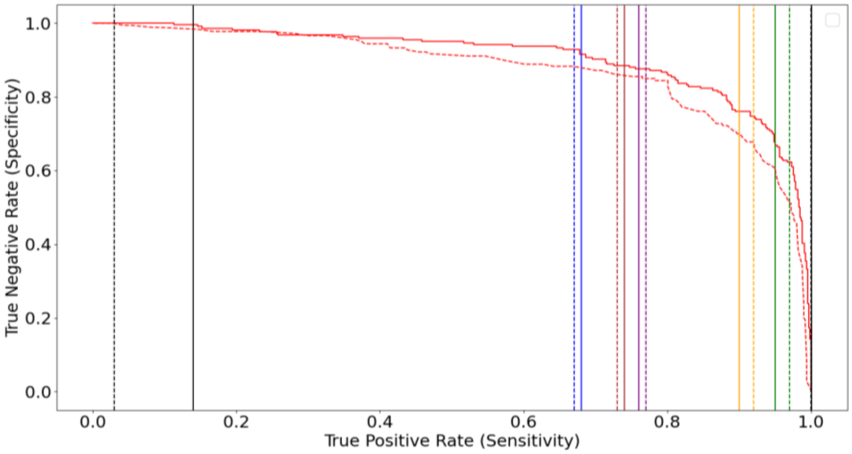

Classification of ≥3 mL HE using CNN+SVM model and thresholds for 100%, 95%, and 90% sensitivity and specificity, as well as the highest accuracy threshold, in the internal test cohort (ATACH-2). These thresholds were then applied to the external validation cohort (Charité). The solid and dashed lines in ROC curve refer to same-color sensitivity/specificity thresholds in internal, and external validation cohorts, respectively.

6 Figure S5. Classification of ≥6 mL HE using CNN+SVM model

≥6 mL HE		Internal test (ATACH-2)		≥6 mL HE		External validation (Charite)	
Accuracy: 18%		**Prediction**		Accuracy: 19%		**Prediction**	
		**– 10 (1.1%)**	**+ 880 (98.9%)**			**- 0 (0%)**	**+ 687 (100%)**
**Ground truth**	–	TN: 10 (1.1%)	FP: 732 (82.3%)	**Ground truth**	–	TN: 0 (0%)	FP: 559 (81.4%)
	+	FN: 0 (0%)	TP: 148(16.6%)		+	FN: 0 (0%)	TP: 128 (18.6%)
**Sensitivity: 100%**		Specificity:1%		Sensitivity: 100%		Specificity: 0%	
Accuracy: 55%		**Prediction**		Accuracy: 40%		**Prediction**	
		**- 359 (40.4%)**	**+ 531 (59.6%)**			**- 161 (23.4%)**	**+ 526 (76.6%)**
**Ground truth**	–	TN: 352 (39.6%)	FP: 390 (43.8%)	**Ground truth**	–	TN: 154 (22.4%)	FP: 405 (59%)
	+	FN: 7 (0.8%)	TP: 141 (15.8%)		+	FN: 7 (1%)	TP: 121 (17.6%)
**Sensitivity: 95%**		Specificity: 47%		Sensitivity: 95%		Specificity: 28%	
Accuracy: 79%		**Prediction**		Accuracy: 62%		**Prediction**	
		**- 580 (65.2%)**	**+ 310 (34.8%)**			**-323 (47%)**	**+364 (53%)**
**Ground truth**	–	TN: 566 (63.6%)	FP: 176 (19.8%)	**Ground truth**	–	TN: 311 (45.3%)	FP: 248 (36.1%)
	+	FN: 14 (1.6%)	TP: 134 (15%)		+	FN: 12 (1.7%)	TP: 116 (16.9%)
**Sensitivity: 91%**		Specificity: 76%		Sensitivity: 91%		Specificity: 56%	
Accuracy: 88%		**Prediction**		Accuracy: 83%		**Prediction**	
		**-851 (95.6%)**	**+39 (4.4%)**			**-677 (98.5%)**	**+10 (1.5%)**
**Ground truth**	–	742 (83.4%)	0 (0%)	**Ground truth**	–	TN: 558 (81.2%)	FP: 1 (0.2%)
	+	109 (12.2%)	39 (4.4%)		+	FN: 119 (17.3%)	TP: 9 (1.3%)
Sensitivity: 26%		**Specificity: 100%**		Sensitivity: 7%		Specificity: 100%	
Accuracy: 92%		**Prediction**		Accuracy: 91%		**Prediction**	
		**- 737 (82.8%)**	**+ 153 (17.2%)**			**-564 (82.1%)**	**+123(17.9%)**
**Ground truth**	–	TN: 705 (79.2%)	FP: 37 (4.2%)	**Ground truth**	–	TN: 531 (77.3%)	FP: 28 (4.1%)
	+	FN: 32 (3.6%)	TP: 116 (13%)		+	FN: 33 (4.8%)	TP: 95 (13.8%)
Sensitivity: 78%		**Specificity: 95%**		Sensitivity: 74%		Specificity: 95%	
Accuracy: 89%		**Prediction**		Accuracy: 85%		**Prediction**	
		**- 652 (73.3%)**	**+ 238 (26.7%)**			**- 460 (67%)**	**+ 227 (33%)**
**Ground truth**	–	TN: 668 (67.2%)	FP: 74 (7.4%)	**Ground truth**	–	TN: 476 (61%)	FP: 83 (12.8%)
	+	FN: 23 (6.1%)	TP: 125 (19.3%)		+	FN: 22 (6%)	TP: 106 (20.2%)
Sensitivity: 84%		**Specificity: 90%**		Sensitivity: 83%		Specificity: 85%	
**Accuracy: 93%**		**Prediction**		Accuracy: 92%		**Prediction**	
		**-748 (84%)**	**+142 (16%)**			**-573 (83.4%)**	**+114 (16.6%)**
**Ground truth**	–	713 (80.1%)	29 (3.3%)	**Ground truth**	–	TN: 537 (78.2%)	FP: 22 (3.2%)
	+	35 (3.9%)	113 (12.7%)		+	FN: 36 (5.2%)	TP: 92 (13.4%)
Sensitivity: 76%		Specificity: 96%		Sensitivity: 72%		Specificity: 96%	

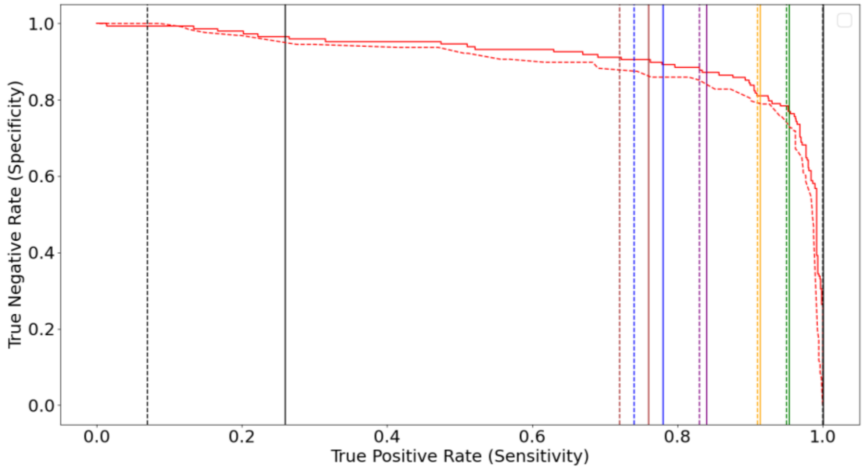

Classification of ≥6 mL HE using CNN+SVM model and thresholds for 100%, 95%, and 90% sensitivity and specificity, as well as the highest accuracy threshold, in the internal test cohort (ATACH-2). These thresholds were then applied to the external validation cohort (Charité). The solid and dashed lines in ROC curve refer to same-color sensitivity/specificity thresholds in internal, and external validation cohorts, respectively.

7 Figure S6. Classification of ≥9 mL HE using CNN+SVM model

**≥9 mL HE**	**Internal test (ATACH-2)**				**≥9 mL HE**	**External validation (Charite)**		
Accuracy: 20%		**Prediction**			Accuracy: 17%		**Prediction**	
		**– 64 (7.2%)**	**+ 826 (92.8%)**				**- 14 (2%)**	**+ 673 (98.0%)**
**Ground truth**	–	TN: 64 (7.2%)	FP: 716 (80.5%)		**Ground truth**	–	TN: 14 (2%)	FP: 569 (82.8%)
	+	FN: 0 (0%)	TP: 110 (12.3%)			+	FN: 0 (0%)	TP: 104 (15.2%)
**Sensitivity: 100%**		Specificity: 8%			Sensitivity: 100%		Specificity: 2%	
Accuracy: 56%		**Prediction**			Accuracy: 35%		**Prediction**	
		**- 401 (45%)**	**+ 489 (55%)**				**- 141 (20.5%)**	**+ 546 (79.5%)**
**Ground truth**	–	TN: 396 (44.5%)	FP: 384 (43.2%)		**Ground truth**	–	TN: 139 (20.2%)	FP: 444 (64.7%)
	+	FN: 5 (0.5%)	TP: 105 (11.8%)			+	FN: 2 (0.3%)	TP: 102 (14.8%)
**Sensitivity: 95%**		Specificity: 51%			Sensitivity: 98%		Specificity: 24%	
Accuracy: 89%		**Prediction**			Accuracy: 71%		**Prediction**	
		**- 704 (79.1%)**	**+ 186 (20.9%)**				**-412 (60%)**	**+275 (40%)**
**Ground truth**	–	TN: 693 (77.9%)	FP: 87 (9.8%)		**Ground truth**	–	TN: 398 (57.9%)	FP: 185 (26.9%)
	+	FN: 11 (1.2%)	TP: 99 (11.1%)			+	FN: 14 (2.1%)	TP: 90 (13.1%)
**Sensitivity: 90%**		Specificity: 89%			Sensitivity: 87%		Specificity: 68%	
Accuracy: 93%		**Prediction**			Accuracy: 90%		**Prediction**	
		**- 842 (94.5%)**	**+ 48 (5.5%)**				**-646 (94.1%)**	**+41 (6.0%)**
**Ground truth**	–	TN:780 (87.6%)	FP: 0 (0%)		**Ground truth**	–	TN:579 (84.3%)	FP: 4 (0.6%)
	+	FN:62 (6.9%)	TP: 48 (5.5%)			+	FN:67 (9.8%)	TP: 37 (5.4%)
**Sensitivity: 44%**		**Specificity: 100%**			Sensitivity: 36%		Specificity: 99%	
Accuracy: 94%		**Prediction**			Accuracy: 86%		**Prediction**	
		**- 757 (85%)**	**+ 133 (15%)**				**-528 (76.9%)**	**+159 (23.1%)**
**Ground truth**	–	TN: 741 (83.3%)	FP: 39 (4.4%)		**Ground truth**	–	TN: 508 (74.0%)	FP: 75 (10.9%)
	+	FN: 16 (1.7%)	TP: 94 (10.6%)			+	FN: 20 (2.9%)	TP: 84 (12.2%)
Sensitivity: 85%		**Specificity: 95%**			Sensitivity: 81%		Specificity: 87%	
Accuracy: 90%		**Prediction**			Accuracy: 73%		**Prediction**	
		**- 714 (80.2%)**	**+176 (19.8%)**				**- 460 (61.6%)**	**+227 (38.4%)**
**Ground truth**	–	TN: 702 (78.9%)	FP: 78 (8.8%)		**Ground truth**	–	TN: 409 (59.6%)	FP: 174 (25.3%)
	+	FN: 12 (1.3%)	TP: 98 (11%)			+	FN: 14 (2%)	TP: 90 (13.1%)
Sensitivity: 89%		**Specificity: 90%**			Sensitivity: 87%		Specificity: 70%	
**Accuracy: 96%**		**Prediction**			Accuracy: 92%		**Prediction**	
		**- 784 (88.1%)**	**+ 106 (11.9%)**				**-583 (84.8%)**	**+104 (15.2%)**
**Ground truth**	–	TN:764 (85.8%)	FP: 16 (1.8%)		**Ground truth**	–	TN:556 (80.9%)	FP: 27 (3.9%)
	+	FN:20 (2.3%)	TP: 90 (10.1%)			+	FN:27 (3.9%)	TP: 77 (11.3%)
Sensitivity: 82%		Specificity: 98%			Sensitivity: 74%		Specificity: 95%	

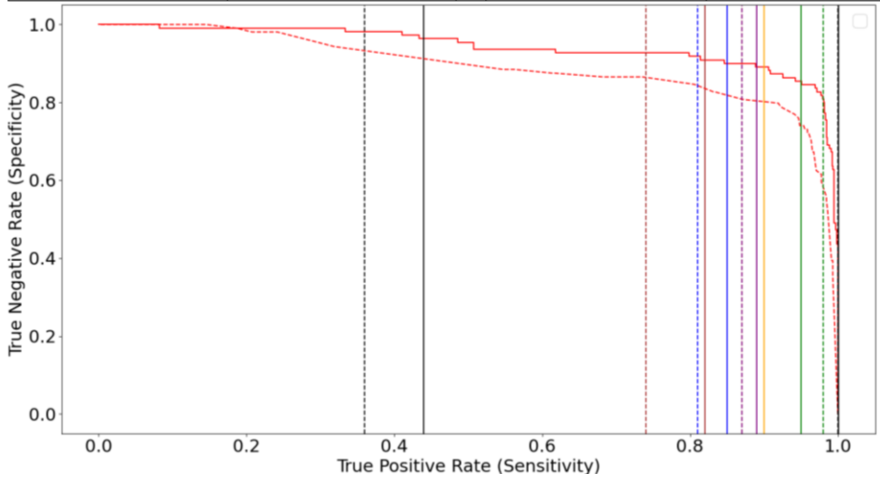

Classification of ≥9 mL HE using CNN+SVM model and thresholds for 100%, 95%, and 90% sensitivity and specificity, as well as the highest accuracy threshold, in the internal test cohort (ATACH-2). These thresholds were then applied to the external validation cohort (Charité). The solid and dashed lines in ROC curve refer to same-color sensitivity/specificity thresholds in internal, and external validation cohorts, respectively.

8 Figure S7. Classification of ≥12.5 mL HE using CNN+SVM model

**≥12.5 mL HE**		**Internal test (ATACH-2)**			**≥12.5 mL HE**		**External validation (Charite)**	
Accuracy: 37%		**Prediction**			Accuracy: 24%		**Prediction**	
		**– 260 (29.2%)**	**+ 630 (70.8%)**				**- 85 (12.4%)**	**+ 602 (87.6%)**
**Ground truth**	–	TN: 260 (29.2%)	FP: 561 (63.0%)		**Ground truth**	–	TN: 83 (12.1%)	FP: 521 (75.8%)
	+	FN: 0 (0%)	TP: 69 (7.8%)			+	FN: 2 (0.3%)	TP: 81 (11.8%)
**Sensitivity: 100%**		Specificity: 32%			Sensitivity: 98%		Specificity: 14%	
Accuracy: 95%		**Prediction**			Accuracy: 82%		**Prediction**	
		**- 780 (87.6%)**	**+ 489 (12.4%)**				**- 511 (74.3%)**	**+ 176 (25.7%)**
**Ground truth**	–	TN: 777 (87.3%)	FP: 44 (5%)		**Ground truth**	–	TN: 495 (72%)	FP: 109 (15.9%)
	+	FN: 3 (0.3%)	TP: 66 (7.4%)			+	FN: 16 (2.3%)	TP: 67 (9.8%)
**Sensitivity: 96%**		Specificity: 95%			Sensitivity: 81%		Specificity: 82%	
ACC=96%		**Prediction**			Accuracy: 89%		**Prediction**	
		**- 799 (89.8%)**	**+ 91 (10.2%)**				**-563 (82%)**	**+124 (18%)**
**Ground truth**	–	TN: 793 (89.1%)	FP: 28 (3.1%)		**Ground truth**	–	TN: 544 (79.2%)	FP: 60 (8.7%)
	+	FN: 6 (0.7%)	TP: 63 (7.1%)			+	FN: 19 (2.8%)	TP: 64 (9.3%)
**Sensitivity: 91%**		Specificity: 97%			Sensitivity: 77%		Specificity: 90%	
Accuracy: 97%		**Prediction**			Accuracy: 93%		**Prediction**	
		**- 845 (95.0%)**	**+ 45 (5%)**				**-639 (93.0%)**	**+48 (7.0%)**
**Ground truth**	–	TN: 821 (92.3%)	FP: 0 (0%)		**Ground truth**	–	TN: 597 (86.9%)	FP: 7 (1.0%)
	+	FN:24 (2.7%)	TP: 45 (5.0%)			+	FN:42 (6.1%)	TP: 41 (6.0%)
**Sensitivity: 65%**		**Specificity: 100%**			Sensitivity: 49%		Specificity: 99%	
ACC: 95%		**Prediction**			Accuracy: 83%		**Prediction**	
		- 785 (88.2%)	+ 105 (11.8%)				**-518 (75.4%)**	**+169(24.6%)**
**Ground truth**	–	TN: 780 (87.6%)	FP: 41 (4.6%)		**Ground truth**	–	TN: 502 (73.1%)	FP: 102 (14.8%)
	+	FN: 5 (0.6%)	TP: 64 (7.2%)			+	FN: 16 (2.3%)	TP: 67 (9.8%)
Sensitivity: 93%		**Specificity: 95%**			Sensitivity: 81%		Specificity: 83%	
ACC=90%		**Prediction**			Accuracy: 71%		**Prediction**	
		- 742 (83.4%)	+ 148 (16.6%)				**- 430 (61.6%)**	**+ 257 (37.4%)**
**Ground truth**	–	TN: 739 (83.1%)	FP: 82 (9.2%)		**Ground truth**	–	TN: 417 (60.7%)	FP: 187 (27.2%)
	+	FN: 3 (0.3%)	TP: 66 (7.4%)			+	FN: 13 (1.9%)	TP: 70 (10.2%)
Sensitivity: 96%		**Specificity: 90%**			Sensitivity: 84%		Specificity: 69%	
**Accuracy: 98%**		**Prediction**			Accuracy: 94%		**Prediction**	
		**- 824 (92.6%)**	**+ 66 (7.4%)**				**-621 (90.4%)**	**+66 (9.6%)**
**Ground truth**	–	TN: 813 (91.4%)	FP: 8 (0.9%)		**Ground truth**	–	TN: 591 (86.0%)	FP: 13 (1.9%)
	+	FN:11 (1.2%)	TP: 58 (6.5%)			+	FN:30 (4.4%)	TP: 53 (7.7%)
Sensitivity: 84%		Specificity: 99%			Sensitivity: 64%		Specificity: 98%	

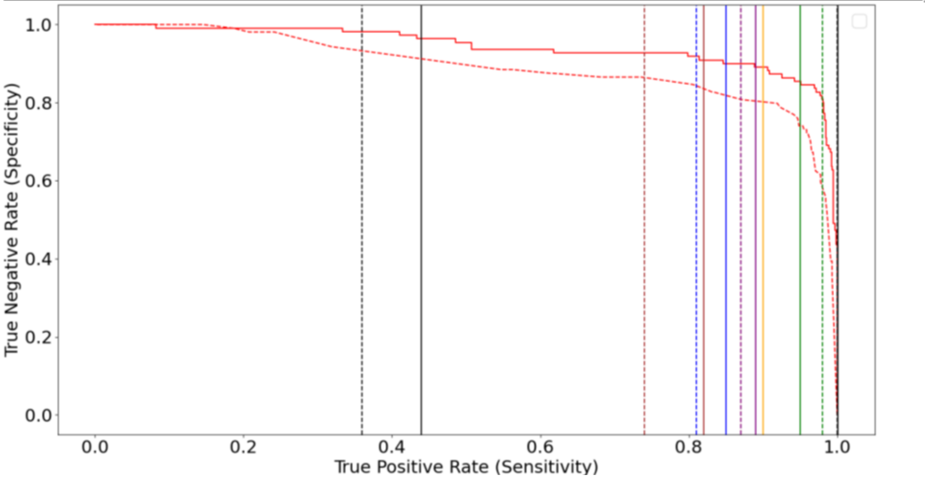

Classification of ≥12.5 mL HE using CNN+SVM model and thresholds for 100%, 95%, and 90% sensitivity and specificity, as well as the highest accuracy threshold, in the internal test cohort (ATACH-2). These thresholds were then applied to the external validation cohort (Charité). The solid

and dashed lines in ROC curve refer to same-color sensitivity/specificity thresholds in internal, and external validation cohorts, respectively.

References

1. R, D.L. Measures of the Amount of Ecologic Association Between Species. *Ecology* **1945**, *26*, 297–302, doi:https://doi.org/10.2307/1932409.

2. Rockafellar, R.T.W., Roger J-B *Variational Analysis.*; Springer-Verlag: 2005.

3. Powers, D.M.W. Evaluation: from precision, recall and F-measure to ROC, informedness, markedness and correlation. *International Journal of Machine Learning Technology* **2011**, *2*, 37-63, doi:10.48550/arXiv.2010.16061.

4. Trevor Hastie, R.T., Jerome Friedman. *The Elements of Statistical Learning: Data Mining, Inference, and Prediction*; 2009.

5. Hanley JA, M.B. The meaning and use of the area under a receiver operating characteristic (ROC) curve. *Radiology* **1982**, *143*, 29-36, doi:10.1148/radiology.143.1.7063747.

6. T, F. An introduction to ROC analysis. *Pattern Recognition Letters* **2006**, *27*, 861-874, doi:10.1016/j.patrec.2005.10.010.

7. Wald, A. Tests of statistical hypotheses concerning several parameters when the number of observations is large. *Transactions of the American Mathematical Society* **1943**, *54*, 426-482.

8. Agresti, A. *An Introduction to Categorical Data Analysis*, 2nd ed.; Wiley-Interscience: 2007.

9. George W. Snecdecor, W.G.C. *Statistical Methods*, 8th ed.; Iowa State University Press: 1989.
